# Expression of PD-L1 and prognosis in breast cancer: a meta-analysis

**DOI:** 10.18632/oncotarget.15532

**Published:** 2017-02-20

**Authors:** Minghui Zhang, Houbin Sun, Shu Zhao, Yan Wang, Haihong Pu, Yan Wang, Qingyuan Zhang

**Affiliations:** ^1^ Department of Medical Oncology, Harbin Medical University Cancer Hospital, Harbin, 150081, China; ^2^ Department of Intervention, Harbin Medical University Cancer Hospital, Harbin, 150081, China; ^3^ Department of Medical Oncology, Heilongjiang Provincial Hospital, Harbin, 150000, China

**Keywords:** meta-analysis, prognosis, programed death-ligand 1, breast cancer

## Abstract

The associations between programmed cell death ligand 1 (PD-L1) and the prognosis of various cancers have always been a research topic of considerable interest. However, the prognostic value of PD-L1 in breast cancer patients remains a controversial subject. We aimed to assess the association between PD-L1 protein expression and clinicopathological features and the impact of this relationship on breast cancer survival. We performed a systematic search of the PubMed, EMBASE, and Cochrane Library databases to determine the correlations among PD-L1 expression, clinicopathological features and overall survival (OS). A total of 5 studies containing 2,546 cases were included in the analysis. The combined hazard ratio (HR) and its 95% confidence interval (CI) for OS were 1.76 (95% CI 1.09–2.82; *P*=0.02) for patients with tumors exhibiting PD-L1 overexpression. The pooled odds ratios (ORs) indicated that PD-L1 expression was associated with positive lymph node metastasis, higher histological grades, estrogen receptor (ER)-negativity, and triple-negative breast cancer (TNBC). Our findings indicate that PD-L1 expression is a promising biomarker for the prognosis of breast cancer, and may be helpful to clinicians aiming to select the appropriate immunotherapy for breast cancer.

## INTRODUCTION

Breast cancer is the most commonly diagnosed cancer and the leading cause of mortality in females worldwide [1]. The incidence of breast cancer has increased steadily in past decades, but the mortality of breast cancer appears to be declining, perhaps as a result of the great progress that has been made in the treatment of breast cancer [2–3]. There are five main treatments for breast cancer, namely, surgery, radiotherapy, chemotherapy, targeted therapy and hormone therapy. However, the efficacies of these therapies in patients with breast cancer remain unsatisfactory due to a lack of effective indicators that can be used to predict disease courses, as well as widespread breast cancer chemo-resistance [4]. Therefore, it is imperative that researchers identify precise biomarkers of breast cancer and potential therapeutic targets for the treatment of the disease to improve survival.

Programmed cell death 1 (PD-1), which belongs to the B7-CD28 superfamily, is a receptor expressed on the surface of T, B and NK cells that regulates their activation and apoptosis [5]. Its ligand, programmed cell death-ligand 1 (PD-L1), is expressed in some tumor cells and by activated B cells and T cells, dendritic cells, macrophages, and fibroblasts cells [6]. PD-L1 binds PD-1 to attenuate the cellular immune response by inducing T-cell apoptosis or exhaustion. Blockade of the PD-1/PD-L1 pathway with monoclonal antibodies (against PD-1 or PD-L1) is a promising therapeutic approach that is currently being explored in studies of many types of human cancer [7]. The results of these studies suggest that PD-L1 plays an important role in tumors immune escapes by facilitating PD-1/PD-L1 pathway activation. PD-L1 expression has been observed in different solid tumors, including breast cancer [8], lung cancer [9], gastric cancer [10], colorectal cancer [11], hepatocellular carcinoma [12], renal cell carcinoma [13], testicular cancer [14] and papillary thyroid cancer [15]. Moreover, several meta-analyses have demonstrated that PD-L1 overexpression signifies a poor prognosis in many cancer types [16–20]. However, the number of studies regarding PD-L1 expression in breast cancer is very limited and the prognostic significance of the protein in breast cancer remains a controversial subject.

To address this issue, we performed meta-analysis to comprehensively evaluate the value of PD-L1 as a prognostic marker, and to determine the relationship between PD-L1 expression and clinicopathological features in breast cancer patients.

## RESULTS

### Search results and study characteristics

**Figure 1:**
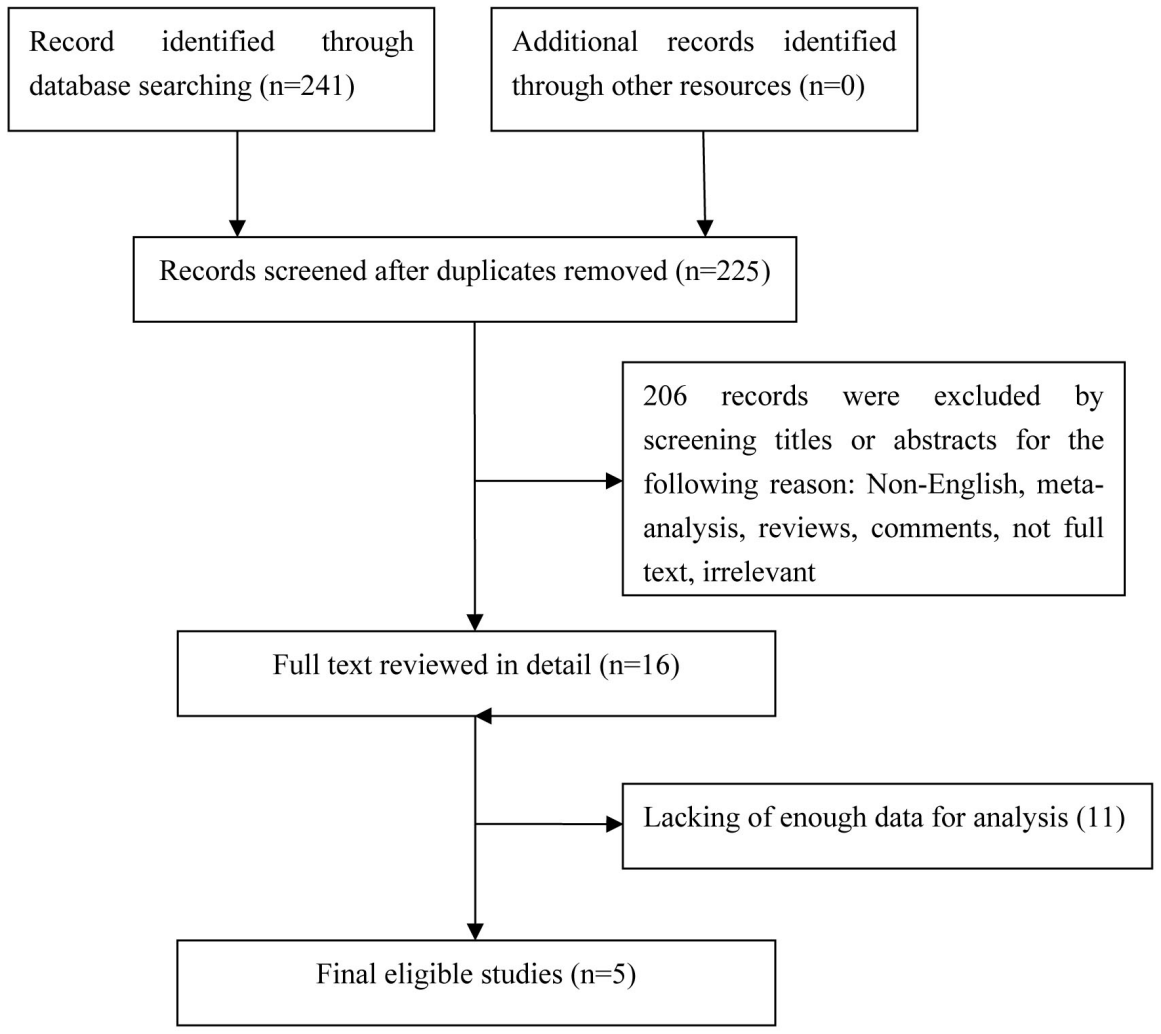
Flow chart of study selection

In this study, we identified a total of 241 potentially relevant articles with our initial search strategy. After screening the titles and abstracts of these articles, we excluded 225 studies because they were duplicates or irrelevant. After reading 16 potentially eligible articles in detail, we determined that 5 trials met our inclusion criteria and thus included these articles in the final analysis. A detailed diagram of the above screening process is presented in Figure 1.

The characteristics of the included studies are presented in Table 1. The sample sizes of these studies ranged from 192 to 870 patients. and a total of 2,546 patients were enrolled in the studies. All 5 of the studies found to be eligible for the analysis were retrospective. Two studies originated from China and the remaining studies originated from Switzerland, Brazil and Korea, respectively. The rates of PD-L1 positivity in the above studies ranged from 21.7% to 56.6%. Four studies featured populations comprising patients with early stage breast cancer. HRs and 95% CIs were obtained directly from all of original articles. All the studies performed IHC analysis to evaluate PD-L1 expression in breast cancer tissues. Study quality, as assessed by Newcastle-Ottawa quality assessment scale, ranged from 6 to 7. Hence, the studies were of a relatively high quality.

**Table 1:** Characteristics of the studies included in the meta-analysis

First Author	Year	Country	NO. of patients	Age, median (range)	IHC evaluation method	Antibody				Cut-off	PD-L1 positive (%)	Follow up Median (range) (M)	Quality assessment (score)
						Company	Source	Type	Clone				
Li	2016	China	501	53 (29-83)	H-score	Abcam, UK	Rabbit	PAB	ab58810	≥100 scores	231/501 (46.1)	64 (1-80)	7
Baptista	2016	Brazil	192	NA	H-score	Abcam, UK	Rabbit	PAB	NA	≥2	107/189 (56.6)	86	6
Muenst	2014	Switzerland	650	64 (27-101)	H-score	Abcam, UK	Rabbit	PAB	M1H1	≥100 scores	152/650 (23.4)	65 (1-174)	6
Park	2016	Korea	333	47 (28-78)	H-score	Abcam, UK	Rabbit	PAB	NA	≥3	163/316 (51.6)	118 (5-154)	6
Qin	2015	China	870	47 (21-84)	Percentage	CST, USA	Rabbit	MAB	NA	≥5%	189/870 (21.7)	98 (17-265)	7

### Association between PD-L1 expression and OS

**Figure 2:**
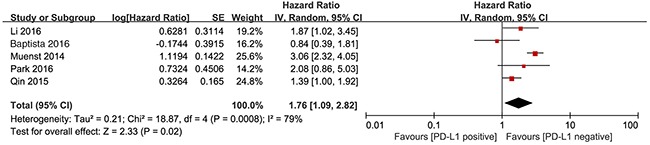
Forest plot describing the association between PD-L1 expression and OS of patients with breast cancer

We investigated the association between PD-L1 expression and OS in breast cancer patients. All 5 studies with a total of 2,546 patients were included. The meta-analysis showed that PD-L1 overexpression was associated with shorter OS in patients with breast cancer than the absence of PD-L1 expression in patients with breast cancer (HR= 1.76, 95% CI 1.09–2.82; *P*=0.02). Significant heterogeneity was observed (I^2^ = 79%, P < 0.01), therefore, a random effects model was used for the analysis (Figure 2).

**Figure 3:**
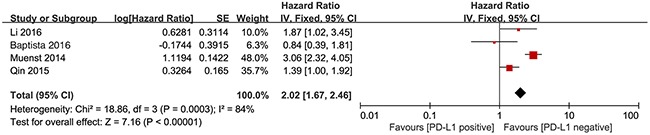
Forest plot describing subgroup analysis of the association between PD-L1 expression and OS after removal of Park et al study

To identify the potential sources of heterogeneity, we performed subgroup analyses. The study by Park et al included only hormone receptor-negative patients. Thus, this study did not include hormone receptor-positive patients. Removal of this study did not significantly affect the association between PD-L1 expression and OS (HR=2.02, 95% CI 1.67-2.46; *P*<0.001) (Figure 3).

### Association between PD-L1 expression and clinicopathological characteristics

**Figure 4:**
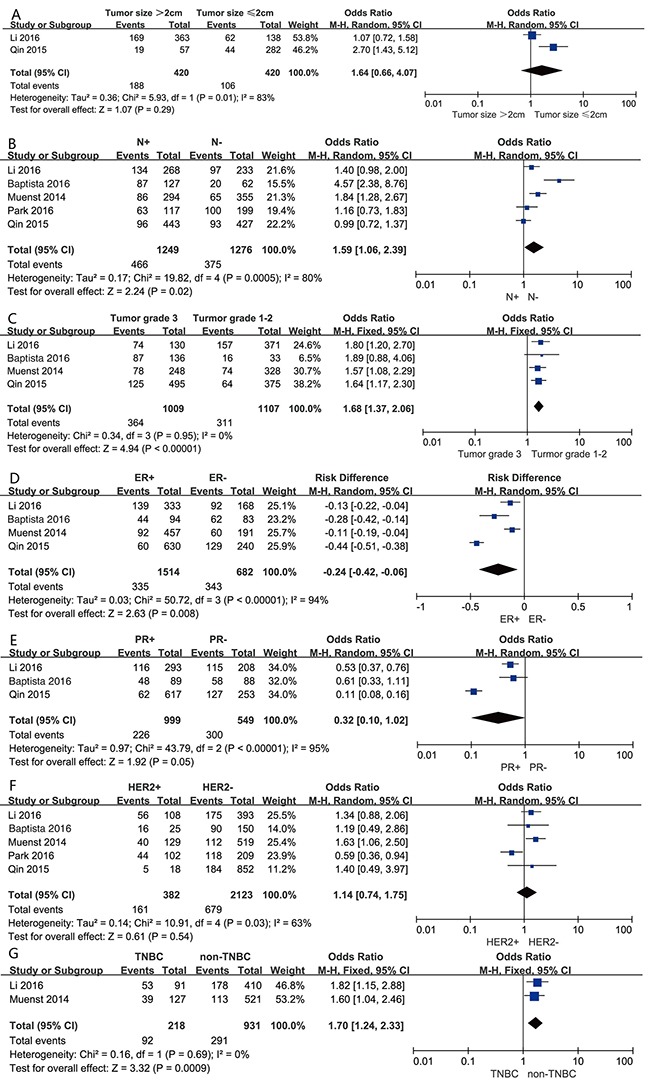
Forest plots for the association between PD-L1 expression and clinicopathological features **(A)** tumor size, **(B)** lymph node metastasis, **(C)** Histological grade, **(D)** ER status, **(E)** PR status, **(F)** HER2 status, **(G)** breast cancer subtypes.

In the present study, we investigated the association between PD-L1 overexpression and clinicopathological characteristics. The pooled results showed that PD-L1 expression was increased in patients with positive lymph node metastasis (OR=1.59, 95% CI 1.06-2.39; *P*=0.02), higher histological grade (OR=1.68, 95% CI 1.37-2.06; P < 0.001), estrogen receptor (ER)-negativity (OR=0.24, 95% CI 0.42-0.06; *P*=0.008) and triple negative breast cancer (TNBC) (OR=1.70, 95% CI 1.24-2.33; *P* < 0.001). However, we detected no significant relationships between PD-L1 overexpression and tumor size (OR=1.64, 95% CI 0.66-4.07, *P*=0.29), progesterone receptor (PR) status (OR=0.32, 95% CI 0.10-1.02; *P*=0.05) or human epidermal growth factor receptor 2 (HER2) status (OR=1.14, 95% CI 0.74-1.75; *P*=0.54) (Figure 4).

Heterogeneity was not observed in the analysis of the relationships between PD-L1 expression and histological grade (*P* =0.95; I^2^=0) and TNBC (*P* =0.69; I^2^=0); thus, a fixed effect model was used. The other analyses were performed using the random effects model.

### Sensitivity analyses

Sensitivity analysis, in which one study was removed at a time, was performed to evaluate the stability of the results. The results of the analysis demonstrated that no individual study significantly influenced the overall HRs, suggesting that the results of the present meta-analysis are credible.

### Publication bias

Egger's and Begg's tests indicated that no publication bias affecting the hazard ratios for OS was present in the included studies. The *P* values for these tests were 0.323 and 0.951, respectively.

## DISCUSSION

PD-L1 overexpression has been observed in various human malignancies, and a previous study has demonstrated that PD-L1 expression contributes to poor prognosis [21]. However, in breast cancer patients, the relationship between PD-L1 expression and prognosis remains unclear. Some studies have shown that positive PD-L1 was associated with significantly poor OS [8, 22–23], but other studies could not confirm this finding [24–25]. The present meta-analysis is the first to investigate the correlation between PD-L1 expression and OS. Our results demonstrate that PD-L1 can serve a significant biomarker in the poor prognosis of breast cancer.

Our findings regarding the adverse effects of increased PD-L1 expression are consistent with those of other studies. For example, in the study by Mao et al [26], which involved 128 non-small cell lung cancer patients, multivariate analyses demonstrated that PD-L1 expression was an independent predictor of poor survival in patients with NSCLC (HR=2.02, 95% CI 1.67-2.46; *P*< 0.001). Moreover, in the study by Eto et al [27], which involved 105 patients with stage II/III gastric carcinoma analysis demonstrated that the 3-year disease-free survival rate was 36.1 % in patients with PD-L1 overexpression and 64.7% in PD-L1-negative patients. Overall survival also tended to be poorer in patients who overexpressed PD-L1 than in patients who did not express PD-L1. In 2016, Roberto et al [20] analyzed the prognostic value of PD-L1 in renal cell carcinoma in a meta-analysis based on 6 studies including 1,323 patients, which demonstrated that positive PD-L1 expression was a negative predictor of OS. However, increased PD-L1 expression has also been reported to be a favorable prognostic factor in patients with NSCLC [28], small cell lung cancer [29], gastric cancer [30], pancreatic cancer [31] and tonsillar cancer [32]. Increased PD-L1 expression was paradoxically associated with improved OS in studies involving patients with the above diseases, possibly because different thresholds were used to determine expression positivity and because the studies included populations of different races. Comparisons of different studies reporting PD-L1 expression in various cancers are hindered by the use of different methodologies, different thresholds, different antibodies and specimens from different areas. Thus, future studies should make an effort to use standardized quantitative assays to measure PD-L1 expression.

Accumulating evidence shows that PD-1/PD-L1 pathway blockade has resulted in sustainable clinical responses and long-term remission in both solid tumors and hematologic malignancies [33]. The efficiency of this approach has varied according to the type of cancer in different clinical trials. In this age of personalized medicine, better immune biomarkers that can predict clinical responses to anti-PD therapy are urgently needed and must be identified and validated in tumor immunotherapy studies. The findings of recent studies indicate that the high PD-L1 expression levels are associated with increased clinical activity in patients with various types of cancer who were treated with PD-1/PD-L1 pathway blockade [34]. Therefore, how patients potentially overexpressing PD-L1 should be screened is a central question faced by researchers attempting to develop anti-PD-1/PD-L1 therapies. In this study, we investigated the relationship between PD-L1 expression and clinicopathological factors. According to the results of our pooled analysis, patients with positive lymph node metastasis, higher histological grades and ER- negativity tended to have higher PD-L1 expression levels than patients without lymph node metastasis, lower histological grades and ER-positivity. The former group of patients may benefit more from treatment targeting the PD-1/PD-L1 pathway. Importantly, we found that PD-L1 was expressed more frequently in TNBC than in non-TNBC. Consistent with this result, recent studies have shown that PD-L1 was expressed mostly frequently in TNBC [35]. A recent study investigated pembrolizumab (a humanized monoclonal antibody against PD-1) safety and antitumor activity in PD-L1-expressing patients with metastatic TNBC. All 32 patients enrolled in the study had previously received chemotherapy. The results of study showed that the objective response rate (ORR) was 18.5% (95% CI 6.3 to 38.1), and the disease control rate(DCR) was 25.9% (95% CI 11.1% to 46.3%) [36]. Taken together, these findings indicate that therapeutic strategies targeting the PD-1/PD-L1 axis are promising for patients with TNBC. In addition, a recent meta-analysis demonstrated that PD-L1 overexpression was significantly associated with positive lymph node metastasis, poor nuclear grades, and ER negativity in breast cancer patients [37]. These results are consistent with those of our study. However, the above study did not show that PD-L1 expression was associated with PR status, HER-2 status, or TNBC. Furthermore, no HRs for OS were included in the analysis, nor was the study by Li et al, which included 501 patients.

We made an effort to conduct a comprehensive analysis, but there were limitations to our study. First, TNM stages and therapeutic strategies, which were not considered in this study, may have impacted on our results. Second, the sample sizes of the studies included in the analysis, as well as the number of studies included in the analysis, were relatively small. However, the results of the sensitivity analysis results remained stable after the sequential exclusion of each individual study. Third, this meta-analysis was limited to articles published in English. In addition, certain studies with negative results may not have been reported, which may have resulted in publication bias. Fourth, PD-L1 positivity was evaluated using different antibodies, and the cutoff values used to determine PD-L1 positivity also varied among the studies included in the analysis. Finally, the quality of the studies included in the analysis may have contributed to the heterogeneity described herein. Despite these limitations, this meta-analysis has demonstrated the correlation between PD-L1 expression and breast cancer clinicopathological factors. The findings of this study may lead to improvements in the outcomes of anti-PD-1/PD-L1 therapy by enabling clinicians to stratify patients in a more appropriate manner. However, despite their robustness, our results regarding, the effectiveness of anti-PD therapy in breast cancer should be interpreted with cautions.

In summary, our results indicate that high PD-L1 expression may be a prognostic indicator for reduced OS. PD-L1 overexpression was significantly associated with a series of clinicopathological parameters, such as large tumor size, lymph node metastasis, and ER-negativity. This information may be helpful to clinicians attempting to screen candidates for anti-PD-1/PD-L1 therapy, especially patients with TNBC. High-quality studies with larger homogeneous populations are needed to determine the role of PD-L1 expression in breast cancer.

## MATERIALS AND METHODS

This meta-analysis was performed according to the preferred reporting items for systematic reviews and meta-analysis (PRISMA) statement [38]. Our study was based on data from previously published studies; therefore, ethical approval was not necessary.

### Literature search

We used the PubMed, EMBASE, and Cochrane databases to perform a comprehensive literature search for published articles. Articles published before April 2016 were included in the anlysis. The following keywords were used for the above searches: (PD-L1 OR B7-H1 OR CD274 OR programmed cell death 1 ligand 1 protein OR CD274 Antigen OR PD-L1 costimulatory protein OR B7H1 Antigen) AND (breast cancer OR breast neoplasms OR breast tumor OR cancer of breast OR human mammary neoplasm OR human mammary carcinomas). To identify additional studies, we also reviewed the reference lists of relevant articles.

### Eligibility criteria

The following studies were included in the analysis: (1) Studies whose entire populations comprised patients with histologically confirmed breast cancer, (2) studies in which PD-L1 expression in breast cancer tissue was detected by immunohistochemistry (IHC), (3) studies providing data regarding the correlation between PD-L1 and clinicopathological features, and overall survival (OS), (4) studies providing sufficient data for the extraction hazard ratios (HRs) and 95% confidence intervals (CIs) for OS, and (5) studies published in English. Studies that failed to meet these inclusion criteria were excluded from the analysis. When duplicate publications were identified, only the most complete or most recent article was included in the analysis.

### Data extraction and quality assessment

All relevant data were extracted by two independent reviewers (ZMH and ZS), and any disagreements were resolved by achieving consensus with the assistance of a third reviewer (SHB). The following information was extracted from each trial included in the analysis: name of the first author, year of publication, country, number of patients, ages of the patients, IHC evaluation methods, antibodies, cut-off values, PD-L1-positivity, follow-up period durations, clinicopathological parameters and HRs and 95% CIs for OS. Quality assessments were conducted independently for each study by two reviewers (SHB and WY) using the Newcastle–Ottawa Quality Assessment Scale (NOS), and any disagreements were resolved by discussion and the achievement of consensus. The NOS maximum possible score is 9 points, and studies that received a score of 6 or higher were considered high-quality studies [39].

### Statistical methods

Pooled ORs and its 95% CIs were used to determine the association between PD-L1 expression and clinicopathological parameters, and HRs and theirs 95% CIs were used to evaluate the association between PD-L1 expression and survival. Heterogeneity among studies was assessed using chi-squared test and I^2^. A P value < 0.1 or an I^2^ statistic >50% was indicative of significant heterogeneity between studies; In these cases, a random-effects model was used. Otherwise, a fixed-effects model was used. Potential publication bias was assessed by Egger's and Begg's test. The meta-analysis was performed with Review Manager 5.3 (Revman the Cochrane Collaboration; Oxford, England) and STATA version 12.0 (Stata Corporation; College Station, TX, USA). P values < 0.05 were considered statistically significant. All P values and 95% CIs were two-sided.

## SUPPLEMENTARY MATERIALS FIGURES AND TABLES






