# Supplementary material for: Expression of PD-L1 and prognosis in breast cancer: a meta-analysis
Source: Oncotarget. 2017 Feb 20;8(19):31347–54. doi: 10.18632/oncotarget.15532 (PMC5458212; doi:10.18632/oncotarget.15532)
Supplement: Supplementary file 1 [file oncotarget-08-31347-s001.pdf]

Expression of PD-L1 and prognosis in breast cancer: a meta-analysis

SUPPLEMENTARY MATERIALS AND METHODS

Appendix 1: Newcastle-Ottawa quality assessment scale

See Appendix File 1

Appendix 2: Search strategies for Pubmed, EMBASE and The Cochrane Library database

See Appendix File 2
