# Supplementary material for: Expression of PD-L1 and prognosis in breast cancer: a meta-analysis
Source: Oncotarget. 2017 Feb 20;8(19):31347–54. doi: 10.18632/oncotarget.15532 (PMC5458212; doi:10.18632/oncotarget.15532)
Supplement: Supplementary file 2 [file oncotarget-08-31347-s002.doc]

**Appendix 1 Newcastle-Ottawa quality assessment scale**

**1.Muenst 2014 (6 socres )**

**Selection**
1) Is the case definition adequate?
a) yes, with independent validation* 1 score
b) yes, eg record linkage or based on self reports
c) no description
2) Representativeness of the cases
a) consecutive or obviously representative series of cases*
b) potential for selection biases or not stated

3) Selection of Controls
a) community controls * 1 score
b) hospital controls
c) no description
4) Definition of Controls
a) no history of disease (endpoint) * 1 score
b) no description of source
**Comparability**
1) Comparability of cases and controls on the basis of the design or analysis
a) study controls for lymph node involvement, tumor grade (Select the most important factor.) *

b) study controls for any additional factor* (This criteria could be modified to indicate specific
control for a second important factor.)
**Exposure**
1) Ascertainment of exposure
a) secure record (eg surgical records) * 1 score
b) structured interview where blind to case/control status*
c) interview not blinded to case/control status
d) written self report or medical record only
e) no description
2) Same method of ascertainment for cases and controls
a) yes * 1 score
b) no
3) Non-Response rate
a) same rate for both groups* 1 score
b) non respondents described
c) rate different and no designation NEWCA

**2. Qin 2015 ( 7 scores)**

**Selection**
1) Is the case definition adequate?
a) yes, with independent validation* 1 score
b) yes, eg record linkage or based on self reports
c) no description
2) Representativeness of the cases
a) consecutive or obviously representative series of cases*
b) potential for selection biases or not stated

3) Selection of Controls
a) community controls * 1 score
b) hospital controls
c) no description
4) Definition of Controls
a) no history of disease (endpoint) * 1 score
b) no description of source
**Comparability**
1) Comparability of cases and controls on the basis of the design or analysis
a) study controls for lymph node involvement, tumor grade, ER status PR status and HER-2 status (Select the most important factor.) * 1 score
b) study controls for any additional factor* (This criteria could be modified to indicate specific
control for a second important factor.)
**Exposure**
1) Ascertainment of exposure
a) secure record (eg surgical records) * 1 score
b) structured interview where blind to case/control status*
c) interview not blinded to case/control status
d) written self report or medical record only
e) no description
2) Same method of ascertainment for cases and controls
a) yes * 1 score
b) no
3) Non-Response rate
a) same rate for both groups* 1 score
b) non respondents described
c) rate different and no designation NEWCA

**3. Li 2016 (7 scores)**

**Selection**
1) Is the case definition adequate?
a) yes, with independent validation* 1 score
b) yes, eg record linkage or based on self reports
c) no description
2) Representativeness of the cases
a) consecutive or obviously representative series of cases*
b) potential for selection biases or not stated

3) Selection of Controls
a) community controls * 1 score
b) hospital controls
c) no description
4) Definition of Controls
a) no history of disease (endpoint) * 1 score
b) no description of source
**Comparability**
1) Comparability of cases and controls on the basis of the design or analysis
a) study controls for lymph node involvement, tumor grade, ER status PR status and HER-2 status (Select the most important factor.) * 1 score
b) study controls for any additional factor* (This criteria could be modified to indicate specific
control for a second important factor.)
**Exposure**
1) Ascertainment of exposure
a) secure record (eg surgical records) * 1 score
b) structured interview where blind to case/control status*
c) interview not blinded to case/control status
d) written self report or medical record only
e) no description
2) Same method of ascertainment for cases and controls
a) yes * 1 score
b) no
3) Non-Response rate
a) same rate for both groups* 1 score
b) non respondents described
c) rate different and no designation NEWCA

**4. Mauricio 2016 (6 scores)**

**Selection**
1) Is the case definition adequate?
a) yes, with independent validation* 1 score
b) yes, eg record linkage or based on self reports
c) no description
2) Representativeness of the cases
a) consecutive or obviously representative series of cases*
b) potential for selection biases or not stated

3) Selection of Controls
a) community controls * 1 score
b) hospital controls
c) no description
4) Definition of Controls
a) no history of disease (endpoint) * 1 score
b) no description of source
**Comparability**
1) Comparability of cases and controls on the basis of the design or analysis
a) study controls for lymph node involvement, tumor grade, ER status PR status and HER-2 status (Select the most important factor.) *
b) study controls for any additional factor* (This criteria could be modified to indicate specific
control for a second important factor.)
**Exposure**
1) Ascertainment of exposure
a) secure record (eg surgical records) * 1 score
b) structured interview where blind to case/control status*
c) interview not blinded to case/control status
d) written self report or medical record only
e) no description
2) Same method of ascertainment for cases and controls
a) yes * 1 score
b) no
3) Non-Response rate
a) same rate for both groups* 1 score
b) non respondents described
c) rate different and no designation NEWCA

**5. Park 2016 (6 scores)**

**Selection**
1) Is the case definition adequate?
a) yes, with independent validation* 1 score
b) yes, eg record linkage or based on self reports
c) no description
2) Representativeness of the cases
a) consecutive or obviously representative series of cases*
b) potential for selection biases or not stated

3) Selection of Controls
a) community controls * 1 score
b) hospital controls
c) no description
4) Definition of Controls
a) no history of disease (endpoint) * 1 score
b) no description of source
**Comparability**
1) Comparability of cases and controls on the basis of the design or analysis
a) study controls for lymph node involvement, tumor grade, ER status PR status and HER-2 status (Select the most important factor.) *
b) study controls for any additional factor* (This criteria could be modified to indicate specific
control for a second important factor.)
**Exposure**
1) Ascertainment of exposure
a) secure record (eg surgical records) * 1 score
b) structured interview where blind to case/control status*
c) interview not blinded to case/control status
d) written self report or medical record only
e) no description
2) Same method of ascertainment for cases and controls
a) yes * 1 score
b) no
3) Non-Response rate
a) same rate for both groups* 1 score
b) non respondents described
c) rate different and no designation NEWCA
