# Supplementary material for: Expression of PD-L1 and prognosis in breast cancer: a meta-analysis
Source: Oncotarget. 2017 Feb 20;8(19):31347–54. doi: 10.18632/oncotarget.15532 (PMC5458212; doi:10.18632/oncotarget.15532)
Supplement: Supplementary file 3 [file oncotarget-08-31347-s003.doc]

**Appendix 2 Search strategies for Pubmed, EMBASE and The Cochrane Library database**

**Pubmed (45)**

1."Breast Neoplasms"[Mesh]

2. Breast Neoplasm[Title/Abstract]

3. Neoplasm, Breast[Title/Abstract]

4. Neoplasms, Breast[Title/Abstract]

5. Tumors, Breast [Title/Abstract]

6. Breast Tumors[Title/Abstract]

7. Breast Tumor[Title/Abstract]

8. Tumor, Breast[Title/Abstract]

9. Mammary Neoplasms, Human[Title/Abstract]

10. Human Mammary Neoplasm[Title/Abstract]

11. Human Mammary Neoplasms[Title/Abstract]

12 .Neoplasm, Human Mammary[Title/Abstract]

13. Neoplasms, Human Mammary[Title/Abstract]

14. Mammary Neoplasm, Human[Title/Abstract]

15. Mammary Carcinoma, Human[Title/Abstract]

16. Carcinoma, Human Mammary[Title/Abstract]

17. Carcinomas, Human Mammary[Title/Abstract]

18. Human Mammary Carcinomas[Title/Abstract]

19. Mammary Carcinomas, Human[Title/Abstract]

20. Human Mammary Carcinoma[Title/Abstract]

21. Breast Cancer[Title/Abstract]

22. Cancer, Breast[Title/Abstract]

23 .Cancer of Breast[Title/Abstract]

24. Mammary Cancer[Title/Abstract]

25. Malignant Neoplasm of Breast[Title/Abstract]

26. Malignant Tumor of Breast[Title/Abstract]

27 .Breast Carcinoma[Title/Abstract]

28. Cancer of the Breast[Title/Abstract]

29.1 or 2 or 3 or 4 or 5 or 6 or 7 or 8 or 9 or 10 or 11 or 12 or 13 or 14 or 15 or 16 or 17 or 18 or 19 or 20 or 21 or 22 or 23 or 24 or 25 or 26 or 27 or 28

30. "Antigens, CD274"[Mesh]

31. CD274 Antigens[Title/Abstract]

32. B7-H1 Immune Costimulatory Protein[Title/Abstract]

33. B7 H1 Immune Costimulatory Protein[Title/Abstract]

34. Programmed Cell Death 1 Ligand 1 Protein[Title/Abstract]

35. CD274 Antigen[Title/Abstract]

36. Antigen, CD274[Title/Abstract]

37. PD-L1 Costimulatory Protein[Title/Abstract]

38. Costimulatory Protein, PD-L1[Title/Abstract]

39. PD L1 Costimulatory Protein[Title/Abstract]

40. Programmed Cell Death 1 Ligand 1[Title/Abstract]

41. B7-H1 Antigen[Title/Abstract]

42. Antigen, B7-H1[Title/Abstract]

43. B7 H1 Antigen[Title/Abstract]

44 .B7H1 Immune Costimulatory Protein[Title/Abstract]

45.30 or 31 or 32 or 33 or 34 or 35 or 36 or 37 or 38 or 39 or 40 or 41 or 42 or 43 or 44

46.29 and 45

((("Breast Neoplasms"[Mesh]) OR (((((((((((((((((((((((((((Breast Neoplasm[Title/Abstract]) OR Neoplasm, Breast[Title/Abstract]) OR Neoplasms, Breast[Title/Abstract]) OR Tumors, Breast[Title/Abstract]) OR Breast Tumors[Title/Abstract]) OR Breast Tumor[Title/Abstract]) OR Tumor, Breast[Title/Abstract]) OR Mammary Neoplasms, Human[Title/Abstract]) OR Human Mammary Neoplasm[Title/Abstract]) OR Human Mammary Neoplasms[Title/Abstract]) OR Neoplasm, Human Mammary[Title/Abstract]) OR Neoplasms, Human Mammary[Title/Abstract]) OR Mammary Neoplasm, Human[Title/Abstract]) OR Mammary Carcinoma, Human[Title/Abstract]) OR Carcinoma, Human Mammary[Title/Abstract]) OR Carcinomas, Human Mammary[Title/Abstract]) OR Human Mammary Carcinomas[Title/Abstract]) OR Mammary Carcinomas, Human[Title/Abstract]) OR Human Mammary Carcinoma[Title/Abstract]) OR Breast Cancer[Title/Abstract]) OR Cancer, Breast[Title/Abstract]) OR Cancer of Breast[Title/Abstract]) OR Mammary Cancer[Title/Abstract]) OR Malignant Neoplasm of Breast[Title/Abstract]) OR Malignant Tumor of Breast[Title/Abstract]) OR Breast Carcinoma[Title/Abstract]) OR Cancer of the Breast[Title/Abstract]))) AND (("Antigens, CD274"[Mesh]) OR ((((((((((((((CD274 Antigens[Title/Abstract]) OR B7-H1 Immune Costimulatory Protein[Title/Abstract]) OR B7 H1 Immune Costimulatory Protein[Title/Abstract]) OR Programmed Cell Death 1 Ligand 1 Protein[Title/Abstract]) OR CD274 Antigen[Title/Abstract]) OR Antigen, CD274[Title/Abstract]) OR PD-L1 Costimulatory Protein[Title/Abstract]) OR Costimulatory Protein, PD-L1[Title/Abstract]) OR PD L1 Costimulatory Protein[Title/Abstract]) OR Programmed Cell Death 1 Ligand 1[Title/Abstract]) OR B7-H1 Antigen[Title/Abstract]) OR Antigen, B7-H1[Title/Abstract]) OR B7 H1 Antigen[Title/Abstract]) OR B7H1 Immune Costimulatory Protein[Title/Abstract]))

**Embase (195)**

1. 'breast tumor'/exp

2. ' Breast Neoplasms ':ab,ti

3. ' Breast Neoplasm ':ab,ti

4. ' Neoplasm, Breast ':ab,ti

5 ' Neoplasms, Breast ':ab,ti

6 ' Tumors, Breast ':ab,ti

7 ' Breast Tumors ':ab,ti

8 ' Breast Tumor ':ab,ti

9 ' Tumor, Breast ':ab,ti

10 ' Mammary Neoplasms, Human ':ab,ti

11 ' Human Mammary Neoplasm ':ab,ti

12 ' Human Mammary Neoplasms ':ab,ti

13 ' Neoplasm, Human Mammary ':ab,ti

14 ' Neoplasms, Human Mammary ':ab,ti

15 ' Mammary Neoplasm, Human ':ab,ti

16 ' Mammary Carcinoma, Human ':ab,ti

17 ' Carcinoma, Human Mammary ':ab,ti

18 ' Carcinomas, Human Mammary ':ab,ti

19 ' Human Mammary Carcinomas ':ab,ti

20 ' Mammary Carcinomas, Human ':ab,ti

21 ' Breast Cancer ':ab,ti

22 ' Cancer, Breast ':ab,ti

23 ' Cancer of Breast ':ab,ti

24 ' Human Mammary Carcinoma ':ab,ti

25 ' Mammary Cancer ':ab,ti

26 ' Malignant Neoplasm of Breast ':ab,ti

27 ' Cancer of the Breast ':ab,ti

28 'Breast Carcinoma ':ab,ti

29.1 or 2 or 3 or 4 or 5 or 6 or 7 or 8 or 9 or 10 or 11 or 12 or 13 or 14 or 15 or 16 or 17 or 18 or 19 or 20 or 21 or 22 or 23 or 24 or 25 or 26 or 27 or 28

30 'programmed death 1 ligand 1'/exp

31 'antigens cd274':ab,ti

32'antigens cd274':ab,ti

33'b7-h1 immune costimulatory protein':ab,ti

34'b7 h1 immune costimulatory protein':ab,ti

35'cd274 antigen':ab,ti

36'antigen cd274':ab,ti

37'pd-l1 costimulatory protein':ab,ti

38'programmed cell death 1 ligand 1':ab,ti

39'b7-h1 antigen':ab,ti

40'antigen, b7-h1':ab,ti

41'b7 h1 antigen':ab,ti

42'b7h1 immune costimulatory protein':ab,ti

43'costimulatory protein, pd-l1':ab,ti

44'pd l1 costimulatory protein':ab,ti

45.30 or 31 or 32 or 33 or 34 or 35 or 36 or 37 or 38 or 39 or 40 or 41 or 42 or 43 or 44

46.29 and 45

**The Cochrane Library [2]**

1. MeSH descriptor: [Breast Neoplasms]

2. Breast Neoplasm:ti,ab,kw

3. Neoplasm, Breast:ti,ab,kw

4. Neoplasms, Breast:ti,ab,kw

5. Tumors, Breast:ti,ab,kw

6. Breast Tumors:ti,ab,kw

7. Breast Tumor:ti,ab,kw

8. Tumor, Breast:ti,ab,kw

9. Mammary Neoplasms, Human:ti,ab,kw

10. Human Mammary Neoplasm:ti,ab,kw

11. Human Mammary Neoplasms:ti,ab,kw

12 .Neoplasm, Human Mammary:ti,ab,kw

13. Neoplasms, Human Mammary:ti,ab,kw

14. Mammary Neoplasm, Human:ti,ab,kw

15. Mammary Carcinoma, Human:ti,ab,kw

16. Carcinoma, Human Mammary:ti,ab,kw

17. Carcinomas, Human Mammary:ti,ab,kw

18. Human Mammary Carcinomas:ti,ab,kw

19. Mammary Carcinomas, Human:ti,ab,kw

20. Human Mammary Carcinoma:ti,ab,kw

21. Breast Cancer:ti,ab,kw

22. Cancer, Breast:ti,ab,kw

23 .Cancer of Breast:ti,ab,kw

24. Mammary Cancer:ti,ab,kw

25. Malignant Neoplasm of Breast:ti,ab,kw

26. Malignant Tumor of Breast:ti,ab,kw

27 .Breast Carcinoma:ti,ab,kw

28. Cancer of the Breast:ti,ab,kw

29.1 or 2 or 3 or 4 or 5 or 6 or 7 or 8 or 9 or 10 or 11 or 12 or 13 or 14 or 15 or 16 or 17 or 18 or 19 or 20 or 21 or 22 or 23 or 24 or 25 or 26 or 27 or 28

30. MeSH descriptor: [Antigens, CD274]

31. CD274 Antigens:ti,ab,kw

32. B7-H1 Immune Costimulatory Protein:ti,ab,kw

B7 H1 Immune Costimulatory Protein:ti,ab,kw

Programmed Cell Death 1 Ligand 1 Protein:ti,ab,kw

35. CD274 Antigen:ti,ab,kw

36. Antigen, CD274:ti,ab,kw

37. PD-L1 Costimulatory Protein:ti,ab,kw

38. Costimulatory Protein, PD-L1:ti,ab,kw

39. PD L1 Costimulatory Protein:ti,ab,kw

40. Programmed Cell Death 1 Ligand 1:ti,ab,kw

41. B7-H1 Antigen:ti,ab,kw

42. Antigen, B7-H1:ti,ab,kw

43. B7 H1 Antigen:ti,ab,kw

44 .B7H1 Immune Costimulatory Protein:ti,ab,kw

45.30 or 31 or 32 or 33 or 34 or 35 or 36 or 37 or 38 or 39 or 40 or 41 or 42 or 43 or 44

46.29 and 45
